# Biocidal Elastic Films Based on Cod Collagen, Pectin, and PMMA Copolymers: Effectiveness in Healing Burn Surfaces in Small Animals

**DOI:** 10.3390/polym18111327

**Published:** 2026-05-27

**Authors:** Veronika Prodaevich, Anna Soloveva, Petr Peretyagin, Evgenia Salomatina, Victoria Rumyantseva, Natalya Valetova, Diana Fukina, Olga Smirnova, Evgeny Suleimanov, Ludmila Semenycheva

**Affiliations:** The Research Institute for Chemistry, Lobachevsky State University of Nizhny Novgorod, pr. Gagarina 23, 603950 Nizhny Novgorod, Russia; prodaevitchnika@yandex.ru (V.P.); sannag5@mail.ru (A.S.); peretyaginpv@gmail.com (P.P.); salomatina_ev@chem.unn.ru (E.S.); vo.rumyantseva@mail.ru (V.R.); nata-bor-2005@mail.ru (N.V.); dianafuk@yandex.ru (D.F.); biodeg@mail.ru (O.S.); suev@mail.ru (E.S.)

**Keywords:** 3D matrix films, cod collagen, pectin, methyl methacrylate, RbTe_1.5_W_0.5_O_6_ complex oxide, photocatalysis, glycerol

## Abstract

This work investigates the conditions for obtaining 3D matrix films for wound dressings based on a copolymer of cod collagen (CC), pectin, and methyl methacrylate (MMA). The synthesis was carried out under photocatalytic conditions using the RbTe_1.5_W_0.5_O_6_ oxide, with glycerol as a plasticizer. The development was based on CC-PMMA-pectin hydrogels synthesized in an aqueous dispersion via photocatalysis in the presence of the complex oxide RbTe_1.5_W_0.5_O_6_. The hydrogels were characterized by elemental analysis and electron microscopy. By mixing the hydrogel and glycerol in a 5:1 ratio, plasticized films (CC-PMMA-pectin-glycerol films) were obtained. These films were transparent, homogeneous, elastic, and easily removed from the polymer substrate. In order to assess the stability of the obtained films, their physical and mechanical characteristics were investigated. An increase in the initial polymer content in the solution from 5% to 12% led to a simultaneous increase in tensile strength (from 0.6 MPa to 0.9 MPa) and elongation at break (from 61% to 76%). It was shown that these films are resistant to bacterial action, which is of great importance for the practical use of such wound dressings in non-sterile conditions. In vivo wound healing tests on rats showed complete wound closure (area reduced to 0 cm^2^ by day 28). Tests of wound healing in rats using new coverings demonstrated high efficiency, providing a rationale for further clinical trials.

## 1. Introduction

The main direction of biomedical polymer chemistry is the development of materials for the pharmaceutical and biotechnological industries, aimed at addressing urgent challenges in modern medicine. One such challenge is the creation of innovative wound dressing materials. These materials play a critical role in wound treatment by providing mechanical protection, stimulating cell proliferation, absorbing exudate, and exerting antimicrobial effects [1]. Skin lesions are a serious problem because wound healing is a complex, multi-phase process. Various dressing materials are employed to support these processes. Currently, there is an active transition from traditional dressings (such as bandages and gauze) to multifunctional hydrogel-based systems. A modern, effective wound dressing must be easy to apply, sterile, antimicrobial, non-adherent, gas-permeable, non-toxic, and have other essential functional properties [2,3,4,5].

Numerous studies have shown that hydrogels, due to their excellent biomedical and mechanical properties, demonstrate promising results in the field of wound healing. The three-dimensional structure of hydrogels is combined with good biocompatibility and the ability to retain large amounts of water and swell without dissolving. When applied to a wound, hydrogels maintain a moist environment and reduce the risk of scarring. They exhibit high absorptive capacity, effectively removing large volumes of exudate from the wound site. Furthermore, the high porosity of hydrogels facilitates oxygen transport. When endowed with biocidal properties, hydrogels protect wounds from fungal and bacterial infections [5,6,7,8,9]. Owing to their varied physico-chemical properties, hydrogel networks can be molded into different sizes and forms. Consequently, hydrogel dressings are highly suitable for the closure of cutaneous wounds [10].

The incorporation of natural polymers into hydrogels promotes the natural healing process, as they serve as an ideal alternative to the extracellular matrix and the original cellular environment of the skin. These polymers may possess additional beneficial properties, such as hemostatic, bacteriostatic, and anti-inflammatory effects [11].

Due to its abundance, simple structure, and biomimetic properties, collagen is widely used in the fabrication of dressing materials to accelerate the wound healing process [12,13,14,15,16,17,18].

Collagen is most commonly subjected to chemical modification by incorporating other polymers to form ionic and intermolecular interactions—most frequently with biopolymers—or by cross-linking macromolecular chains using specific agents such as genipin, glutaraldehyde (GA), etc. To achieve the desired characteristics, polysaccharides and synthetic polymers are introduced into these collagen-based composite formulations through various methods. This results in enhanced physicochemical properties of the materials, such as improved mechanical strength, stability, and denaturation temperature, as well as an overall superior healing effect [19]. Unlike many other traditional systems in which biocidal additives are introduced into the matrix separately, for example silver nanoparticles (AgNPs) [20] and ZnO nanoparticles [21], the RbTe_1.5_W_0.5_O_6_ photocatalyst is used in our work, which has its own pronounced bactericidal activity and ensures the formation of a polymer network.

Previous work [22] presented the results of the synthesis and small animal testing of wound dressings in the form of sponge-like plates based on a cod collagen and MMA copolymer, incorporating pectin, polyethylene glycol, and organic cross-linking agents as modifiers. Wound healing trials in rats demonstrated significantly higher efficiency of these new coatings compared to commercial bovine collagen-based samples. The application of the cod collagen-based dressing facilitated the normalization of microcirculation levels, as confirmed by laser Doppler flowmetry, as well as a rapid reduction in the area of scalded burn wounds according to planimetric data. These effects may be attributed to the improvement of trophic processes within the dermal layers. The pro-regenerative properties of the investigated coatings were manifested in a significant acceleration of the healing of experimental thermal skin burns.

The effective performance of these sponge plates in animal models served as a starting point for further research aimed at expanding the range of wound dressings. The coatings described in [22] are water-saturated sponge plates with biocidal properties, allowing them to be stored and applied without additional sterilization when kept in distilled water. However, in their dried state, they form brittle films that are inconvenient for storage. In contrast, elastic films with a three-dimensional structure, which transform into hydrogels upon contact with water, are significantly more practical for clinical use [23,24].

The purpose of this work (1) is to create elastic films with a three-dimensional structure based on cod collagen, pectin, and PMMA copolymers synthesized via heterogeneous photocatalysis in an aqueous dispersion of the complex oxide RbTe_1.5_W_0.5_O_6_ under visible light irradiation, and (2) to evaluate their effectiveness in healing burn surfaces in small animals.

## 2. Materials and Methods

### 2.1. Materials

Commercial reagents were used: apple pectin (Xlebzernoprodukt, Taganrog, Russia), acetic acid (pure for analysis, Lega, Dzerzhinsk, Russia), sodium hydroxide (pure for analysis, Reahim, Moscow, Russia), and glycerin (pure for analysis, Vecos, Nizhniy Novgorod, Russia). MMA (pure for analysis, Sigma Aldrich, St. Louis, MI, USA) was pre-purified from the stabilizer by sequential washing with a sodium hydroxide solution and cold water until a neutral pH was reached; it was then dried over anhydrous calcium chloride and distilled under reduced pressure. The RbTe_1.5_W_0.5_O_6_ photocatalyst was synthesized via the solid-state method [25]. Collagen was isolated by acetic acid extraction for 24 h at room temperature, according to the procedure described in [26]. The resulting acetic acid dispersion was dried to a constant weight in a vacuum (1.33 Pa) at 50 °C.

### 2.2. Synthesis and Drying of the Copolymer

**Figure 1:**
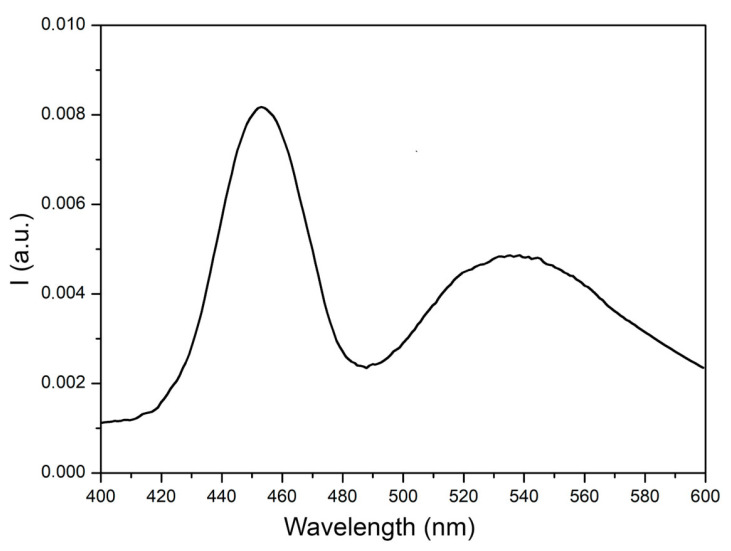
Lamp spectrum (white, 30 W LED, 6500 K) [27].

Pectin and collagen were dissolved in water at a 50:50 ratio at 45 °C under stirring. The RbTe_1.5_W_0.5_O_6_ catalyst (λ = 400–700 nm) in a catalyst:solution ratio of 1:170 of the initial components, the pectin-collagen solution, and MMA were transferred into a flat-bottomed conical flask with a side neck, and the required amount of water was added. Prior to synthesis, the solution was bubbled with argon for 15 min. The synthesis was carried out under the influence of two LED daylight lamps (power 30 W, radiation 400–600 nm, Figure 1) with argon purging and constant magnetic stirring for 4 h. After the synthesis, the catalyst was separated by centrifugation at 5000 rpm for 30 min on a RHON-6000E laboratory centrifuge (Redhon, Saint-Petersburg, Russia). The polymer was dried under vacuum (1.33 Pa) to a constant weight at 40 °C.

### 2.3. Elemental Microanalysis (CHNS Analysis)

The elemental composition of the pectin was determined using a Vario EL cube universal elemental analyzer with simultaneous determination of CHNS (Elementar Analysensysteme GmbH, Langenselbold, Germany).

### 2.4. Sample Preparation for a Scanning Electron Microscope (SEM)

To obtain a sponge, the copolymer was freeze-dried. The initial polymer was dried to a constant weight, after which an aqueous solution with a concentration of 50% was prepared. The copolymer solution was transferred to a round-bottomed flask, frozen with liquid nitrogen (−196 °C) and dried under vacuum (1.33 Pa) for three hours. Drying was carried out using a Vacuubrand MZ 2C NT chemical diaphragm pump (Vacuubrand GMBH + CO KG, Wertheim, Germany, capacity 2.0 m^3^/h).

### 2.5. SEM

The structure was studied using a JSM-IT 300 scanning electron microscope (Jeol Ltd., Tokyo, Japan) with an electron probe diameter of 5 nm (operating voltage 20 kV) using detectors of low-energy secondary electrons and backscattering electrons in a low vacuum mode to avoid charging the samples.

### 2.6. Investigation of Physical and Mechanical Properties

The tensile strength and deformation of glycerin-modified and air-dried hydrogels were studied on a universal bursting machine SUBRAMAX (SUBRA LLC, Neftekamsk, Russia) at a strain rate of 10 mm/min with automatic data recording. Ten film samples of each type, with dimensions of 50 × 10 mm, were tested. The thickness was measured using a micrometer. The obtained values for tensile strength and elongation at break were averaged.

### 2.7. Bactericidal Activity Assay

For the experiment, an association of test cultures of bacteria with high hydrolytic activity was used: *Escherichia coli* ATCC 2592, *Staphylococcus aureus* ATCC 6538, and *Bacillus subtilis* ATCC 6633. The association of bacteria most commonly found in the environment and belonging to various types of bacteria was used: gram− (*E. coli*), gram+ (*S. aureus*) and spore-forming bacteria with high resistance in the external environment. This is due to the fact that laboratory tests are as close as possible to natural conditions, where, as a rule, the biodegradation of industrial materials is carried out by several types of bacteria and in most cases, this process can be simultaneous. For the experiment, bacterial test cultures were resuspended in sterile distilled water. The initial titer of the suspension was 1 × 10^6^ cells/mL. Samples of a 50 × 10 mm collagen copolymer containing submicron particles of complex oxide RbTe_1.5_W_0.5_O_6_ were placed in Petri dishes on the surface of GRM agar inoculated with a bacterial suspension (0.1 mL). Cultivation was carried out in a thermostat at a temperature of 37 °C for 24 h. Some of the samples were exposed to light under these conditions, and some remained in the dark. To control the viability of bacteria, a lawn of their association was sown on the surface of an agarized medium (GRM) in Petri dishes without the presence of samples. Next, we evaluated the bactericidal properties of the samples (Table 1).

In a number of international standards for assessing the bactericidal activity of polymer materials, it is assessed by the presence of a bacterial growth inhibition zone around the sample (ISO 846:2019) [28]. In most cases, depending on the diameter of the bacterial growth inhibition zone, biocidal formulations are classified as non-bactericidal (up to 5 mm), mildly bactericidal (from 5 to 8 mm), bactericidal (from 9 to 19 mm) and strongly bactericidal (over 19 mm) [29].

The light source was a dust- and moisture-proof LED floodlight JAZZWAY PFL-C3 (50 W). The flux density of radiation acting on the surface of the samples was 524 W/m^2^.

All the results obtained in three independent experiments, in 3 repetitions, were processed using the program Statistica 10.0 and Microsoft Excel 2007. The validity of the differences in the mean values was assessed according to Student’s criterion for a probability level of at least 95%.

### 2.8. Wound Healing Study on a Thermal Burn Model

The experiment was conducted on 40 male Wistar rats weighing 250–300 g. Prior to the start of the experiment, all individuals were kept in quarantine for 14 days. The conditions of housing corresponded to the standards prescribed in the “Sanitary rules for the establishment, equipment and maintenance of experimental biological clinics” (departmental acts of the USSR Ministry of Health No. 1045 dated 4 June 1973 and No. 1179 dated 10 October 1983): standard food ration, free access to water, natural lighting, and temperature range of 18–22 °C. The study was approved by the Bioethical Committee of Lobachevsky University (Protocol No. 101 dated 15 July 2025). The work was carried out in accordance with the principles of humanity set out in the Directives of the European Community (No. 86/609/EEC, Strasbourg, 1986) and the Helsinki Declaration (2000).

Thermal damage to the skin of the second degree with an area of 10% of the entire body surface was created on the previously depilated area of the back. For this, combined anesthesia was used (Zoletil 100 (60 mg/kg) + Xylavet (6 mg/kg)), and a steel plate template heated to 240 °C was pressed against the skin for 5 s [30].

The wounds were treated using a closed method. Sterile hydrogel bandages, Cosmopor E coating (Hartmann, Heidenheim, Germany) and self-adhesive Peha-haft bandages (Hartmann, Heidenheim, Germany) were used. The test material was placed between the wound surface and the hydrogel layer. The group of animals without treatment lacked the test material, but the remaining layers of the dressing were preserved (hydrogel bandages, Cosmopor E coating, and self-adhesive Peha-haft bandages). To ensure humane treatment and prevent infectious complications that could lead to premature death of animals or systemic damage, all groups of animals (including negative controls) were daily soaked in a bandage with a 4% gentamicin solution. This regime corresponds to the standard veterinary practice of treating burn wounds in rodents. Antibiotics were used equally in all groups, which makes it possible to compare different wound dressings. However, the presence of gentamicin does not allow us to evaluate the biocidal activity of films in vivo, so the discussion of antibacterial properties is limited to in vitro data (Table 2). Bandages were changed every 3–4 days using general anesthesia (Zoletil 100 (60 mg/kg) + Xylavet (6 mg/kg)).

Animals were removed from the experiment on the 28th day under anesthesia (Zoletil 100 (60 mg/kg) + Xylavet (6 mg/kg)). Intermediate assessments were performed on days 7, 14, and 21.

All rats were randomly divided into four groups of ten individuals each: Group 1—intact rats; Group 2—negative control, including animals with a natural course of the wound process without any treatment nor local applications; Group 3—positive control, where a ready-made collagen wound coating was applied to the wound (ZAO Zelenaya Dubrava, Dmitrov, Russia); Group 4—experimental, where the damaged area was treated with the studied biocidal elastic films.

The planimetric method was used to assess the healing rate of the burn defect. Starting from the second day after the injury, the contours of each defect were transferred daily to a transparent film, and then the area was calculated using graph paper. The area (S, cm^2^) of the superficial skin defect was calculated using the formula S = n + ½ k (1), where n is the number of whole 1 × 1 mm squares lying completely inside the wound contour, and k is the number of 1 × 1 mm squares partially overlapping the wound contour [31].

For histological analysis, skin samples were fixed in 10% neutral formalin. Serial slices 4–6 microns thick were prepared from paraffin blocks (KHIMPEC, Saint-Petersburg, Russia) on the MSE electronic microtome (Inmedprom LLC, Yaroslavl, Russia). The sections were stained with hematoxylin (HiMedia Laboratories, Mumbai, India) and eosin Y (Panreac AppliChem, Barcelona, Spain) [30]. The preparations were viewed and micrographed using the Leica DM 1000 morphometric system (Leica Microsystems, Wetzlar, Germany), to which a Leica DFC 290 HD digital camera (Leica Microsystems, Germany) is connected.

The level of microcirculation in the intact skin and the burn area was assessed by laser Doppler flowmetry (LDF) [32] using a LACK-M laser analyzer (Lazma, Moscow, Russia). During the study, the analyzer probe was installed perpendicular to the area under study. Microcirculation of the skin of the marginal area of the burn in the interscapular space on the right, at a distance of 1 cm from the spine, was assessed. A similar area was studied in healthy animals. The recording duration was 3 min. The assessment of the blood flow status was carried out immediately after the injury and on the 7th, 14th, 21st and 28th days of the experiment. When analyzing tissue microhemocirculation, the microcirculation index (PM) was calculated using the formula PM = K × N_er_ × v_av_ (2), where K = 1 (proportionality coefficient), n_er_ is the number of red blood cells, and v_av_ is the average velocity of red blood cells in the volume under study. This indicator reflects the average perfusion level (the average flow of red blood cells) per unit volume of tissue per unit time.

The LDF 3 software was used to perform a wavelet analysis of blood flow frequency fluctuations in order to identify the contribution of active regulatory mechanisms (endothelial fluctuations (E) range 0.01–0.08 Hz; neurogenic (N) range 0.08–0.2 Hz; myogenic (M) range 0.2–0.7 Hz) and passive factors (respiratory fluctuations (R)—0.7–2 Hz; cardiac (C)—2–5 Hz).

### 2.9. Statistical Analysis

Statistical processing of the obtained data was performed using Microsoft Excel and Statistica 6.0 (Statsoft Inc., Tulsa, OK, USA) software packages. The results of the experimental study are presented as the arithmetic mean and standard deviation (M ± σ). Before conducting intergroup comparisons, the distribution of the data was assessed using the Shapiro–Wilk test. The significance of intergroup differences was evaluated using Student’s *t*-test with Bonferroni correction for multiple comparisons. Differences were considered statistically significant at *p* < 0.05.

## 3. Results

**Figure 2:**
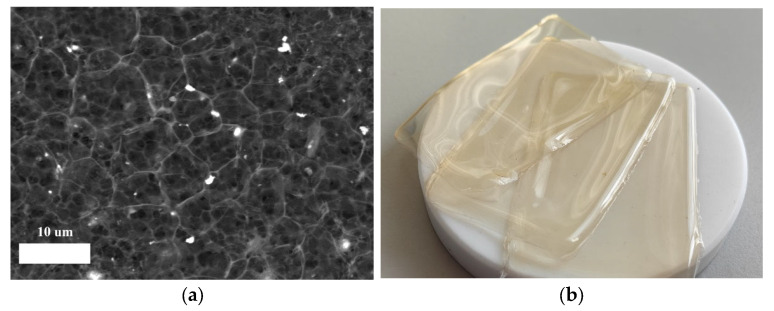
SEM image of the CC-PMMA-pectin copolymer (**a**) [33]; photograph of the wound dressing: CC-PMMA-pectin-glycerol film (**b**).

The elastic films in this study were developed based on CC-PMMA-pectin hydrogels obtained in an aqueous dispersion via photocatalysis in the presence of the complex oxide RbTe_1.5_W_0.5_O_6_. They were characterized by elemental analysis and electron microscopy. According to the results of the elemental analysis, in the initial mechanical mixture of pectin and collagen (the ratio is 50:50 wt.%), the nitrogen content is 7%, whereas in the copolymer it is 5.6%. The conversion method was used to determine the content of CC in the copolymer with pectin and MMA as 40%. The SEM image of the lyophilized sample revealed the formation of a fine-mesh porous matrix that is easily filled with water to form a hydrogel (Figure 2). These hydrogel samples were used for wound healing applications. It was shown that these new materials are resistant to fungi, which is crucial for the long-term functional performance of the specific product under conditions of potential fungal exposure [33].

**Figure 3:**
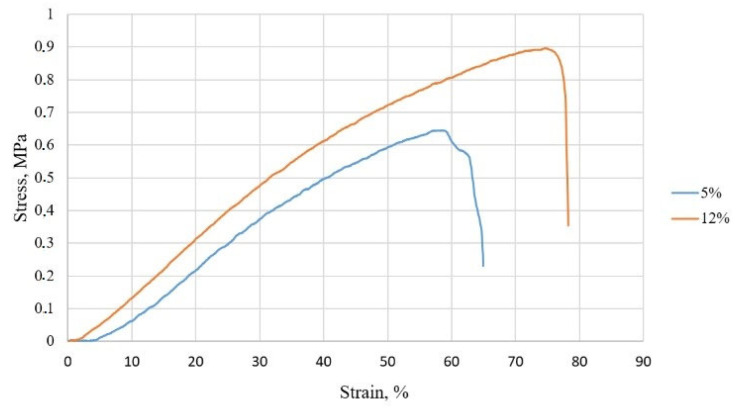
Representative stress–strain curves for elastic polymer films with an initial polymer content in solutions of 5 and 12%.

In previously conducted studies, biocompatible polyols such as glycerol, propylene glycol, dipropylene glycol, and others have most frequently been used as plasticizers for natural polymers [34,35,36,37]. Glycerol has been investigated more often than others, yielding successful experimental results. Consequently, in the present work, films were prepared using glycerol. A solution of the polymer CC-PMMA-pectin obtained by photocatalysis in the presence of a complex oxide RbTe_1.5_W_0.5_O_6_ [33] was mixed with glycerin to obtain elastic films. After drying the polymer to a constant weight, it was re-dissolved in water at concentrations of 5 and 12% at a 5:1 ratio of polymer solution:glycerin. Films with glycerin plasticizer (CC-PMMA-pectin-glycerin films) were obtained, which were transparent, homogeneous, elastic and easily removed from the polymer substrate (Figure 2). In order to assess the stability of the obtained films, their physical and mechanical characteristics were investigated. An increase in the initial polymer content in the solution from 5% to 12% led to a simultaneous increase in tensile strength (from 0.6 MPa to 0.9 MPa) and elongation at break (from 61% to 76%) (Figure 3).

From the presented data, it can be concluded that the low strength of the films is due to the presence of a sufficiently large amount of plasticizer in the form of glycerin and water, since with a decrease in the water content in the initial solution, the physico-mechanical properties of the films improve. For example, in a study (Jiao. J et al.) [38] using the example of composite films made of silk fibroin and sodium alginate with different glycerol contents, it was shown that the more glycerol in the composition, the lower their strength and the higher their ability to reverse deformation. The results of the study of camel gelatin samples in different age groups of animals showed that the age of camel gelatin, as well as the presence of glycerin and sorbitol, as plasticizers, have a significant effect on the properties of the film. The tensile strength ranged from 0.32 MPa to 3.99 MPa, and the relative strain at break ranged from 89.42% to 2.68% [39].

It should be noted that while the mechanical properties of wound dressings are important, they play a secondary role compared to other functional characteristics such as biocompatibility and porosity, which we investigated previously [33], as well as biocidal properties, which were studied in view of the potential use of these new films as wound coverings. The bactericidal activity was determined against a consortium of test bacterial cultures: *Escherichia coli*, *Staphylococcus aureus*, and *Bacillus subtilis*. The use of associations of bacterial cultures, rather than individual species, is widely used, particularly in assessing resistance to bacterial action such as fuels, oils, lubricants, and coolants [40,41,42]. The results of the bactericidal activity studies are presented in Table 1.

It can be seen that under light irradiation (50 W), the CC-PMMA-pectin-glycerol films exhibited strong bactericidal properties, whereas samples not exposed to light showed only weak bactericidal activity against the consortium of test bacterial cultures. This is likely due to the ability of the catalyst particles to interact with the visible light spectrum, thereby inducing photochemical reactions that generate reactive oxygen species (ROS) [43], which enhance the antimicrobial effect of the particles.

The biocidal properties of these films are of great importance for wound dressings. Firstly, they can be utilized under non-sterile conditions. Secondly, the development of wound dressings must address the issue of antibiotic resistance—the ability of bacteria to survive and multiply despite the presence of antibiotics that previously effectively eliminated these microorganisms [24,43,44,45,46]. Although the in vitro bactericidal activity of the CC-PMMA-pectin-glycerol films under visible light was clearly demonstrated (Table 2), the in vivo wound healing experiment was conducted with daily application of 4% gentamicin as a standard antibiotic prophylaxis to avoid uncontrolled infection across all groups. Therefore, the observed acceleration of healing cannot be solely attributed to the biocidal properties of the films; rather, it reflects their pro-regenerative capacity (microcirculation recovery) against a background of adequate antimicrobial protection. The potential additive or synergistic antimicrobial effect of the films in vivo requires further investigation without concomitant antibiotic usage.

The efficacy and safety of the CC-PMMA-pectin-glycerol films were evaluated in the treatment of burn wounds in small animals (rats). The animal experiments were conducted in compliance with humanitarian principles, following the European Community Directives (No. 86/609/EEC, Strasbourg, 1986) and the Declaration of Helsinki (2000). The animals were housed under standard vivarium conditions in cages with *ad libitum* access to food and water, in accordance with the GOST standards for the “Maintenance of experimental animals in research institutes.”

Previously, we investigated wound dressings in the form of sponge-like plates based on a cod collagen-MMA copolymer modified with pectin, polyethylene glycol, and organic cross-linking components for the healing of burn surfaces in rats. Those wound healing trials demonstrated significantly higher efficacy compared to both commercial bovine collagen-based products and untreated control groups [22].

**Figure 4:**
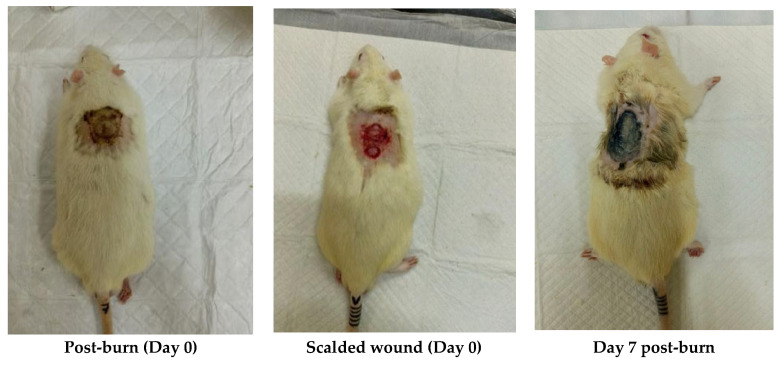

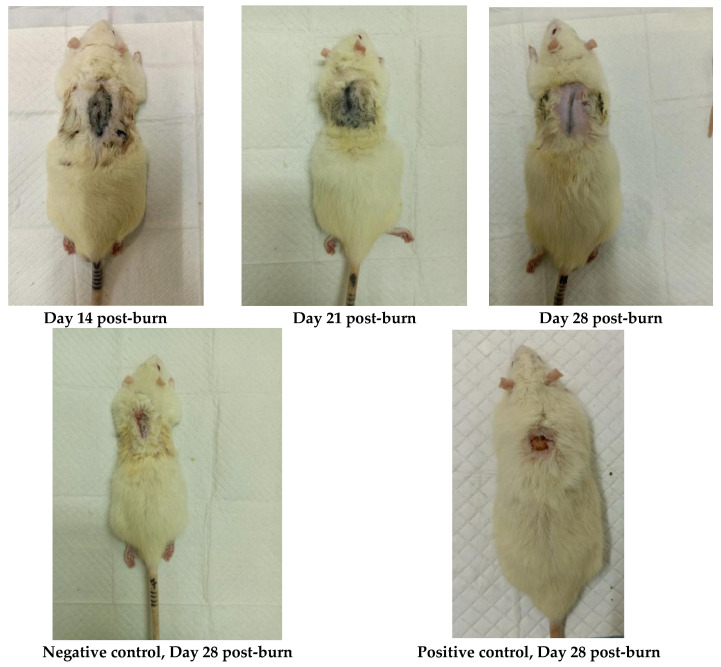
Skin regeneration in the experimental group.

In the present work, the CC-PMMA-pectin-glycerol hydrogel film was applied to the affected area under similar conditions, and the healing process was monitored over 28 days. Consistent with the trials described in [22], animal activity was reduced during the first 24 h post-burn modeling, with food intake occurring in small portions. In the experimental group, motor activity and appetite were fully restored by the third day after the burn injury, similar to the results observed with the sponge-like plates. The regeneration process of burn wounds in rats treated with the CC-PMMA-pectin-glycerol film is presented in Figure 4.

Contour assessment of the area of thermal skin damage on the 7th day of the experiment revealed that the absolute values of this parameter in the experimental group were slightly better than those for the collagen-based sponge plates (coating 1—sponge plate based on the CC-PMMA-PEG copolymer; coating 2—sponge plate based on the CC-PMMA-pectin copolymer [22] (Table 2)). Accordingly, a significantly higher efficacy was demonstrated compared to spontaneous wound healing without treatment.

Meanwhile, the rate of the scab area decreased by the 7th day in the experimental group amounted to 22.5%. Darkly colored particles appeared on the scab surface and persisted until the end of the experiment. Histological examination of the skin flap section showed that it was unevenly infiltrated with melanophages, which caused the dark color of the lesion.

The higher recovery rates in the experimental group at this regeneration stage can be explained by morphological changes: rapid relief of inflammation and no tendency of the inflammatory infiltrate to spread into surrounding tissues. All of this characterizes the wound field cleansing process as higher quality and more intensive and leads to the transition from the inflammation stage to the stage of proliferation, which is characterized by the formation of granulation tissue in the wound area.

The areas of thermal skin defects continued to shrink at an identical rate in the animals from the compared groups on both the 14th and 21st days of the experiment (Table 2).

By the 28th day of the experiment, the burn area in the experimental group rats, similar to spongy plates, consisted of connective tissue scars and in some places looked like undamaged skin; hair regrowth had started (Figure 4). This indicates the nearly complete termination of regenerative processes under the influence of the investigated coatings.

**Figure 5:**
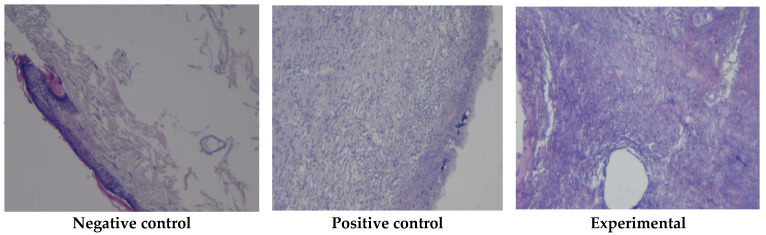
Histological appearance of the rat skin flap section.

The histological appearance of the skin flap section from a rat treated with the experimental coating (Figure 5) was characterized by mixed-cell inflammation, which was observed in the dermis. The entire flap was unevenly infiltrated with melanophages. The cells were discrete, with no specific abnormalities. No evidence of bacterial infection or neoplasia was detected. So, the detected tissue changes are consistent with regenerative processes, which involve epithelialization, scar tissue formation, and hyperpigmentation.

**Figure 6:**
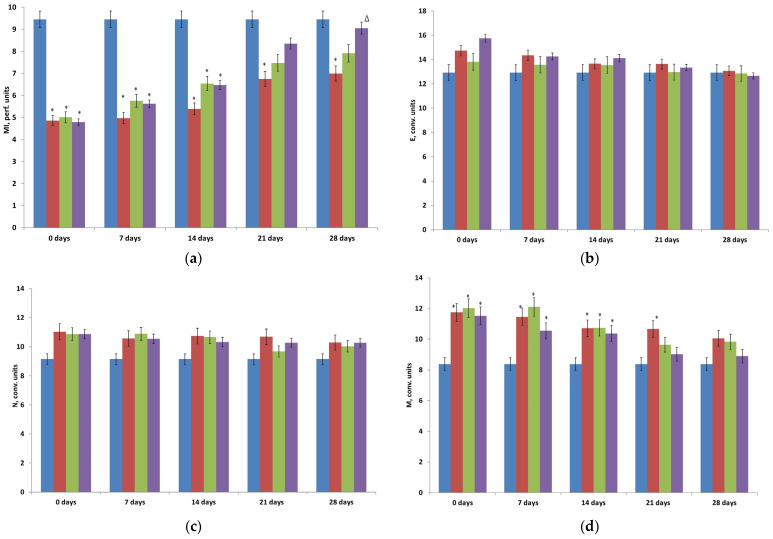

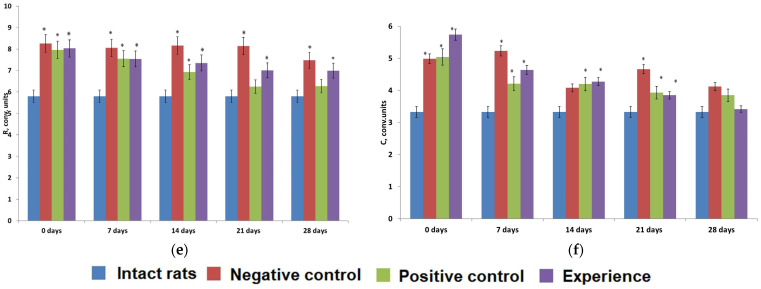
State of microcirculation during thermal injury with the use of biocidal elastic films, where (**a**)—microcirculation index (MI); (**b**)—endothelial oscillations (E); (**c**)—neurogenic oscillations (N); (**d**)—myogenic oscillations (M); (**e**)—respiratory oscillations (R); (**f**)—cardiac oscillations (C) of microvascular blood flow regulation factors; *—differences are statistically significant compared to intact rats (*p* < 0.05); Δ—differences are statistically significant compared to control 1 (*p* < 0.05).

To evaluate the effectiveness of tissue perfusion restoration against the background of the use of the developed biocidal elastic films (group 4), laser Doppler flowmetry (LDF) was performed during the experiment (days 0, 7, 14, 21, and 28). The comparison was performed with intact animals (group 1), negative controls (without treatment, group 2), and positive controls (commercial collagen coating, group 3). The main results are shown in Figure 6a–f.

Microcirculatory index (MI, Figure 6a) was assessed. In intact animals, the MI was stable throughout the study (9.46 ± 0.8 perf. units). On day 0 (immediately after the burn), a sharp decrease in MI to 4.8–5.0 perf. units was observed in all experimental groups, which corresponded to pronounced vasospasm and ischemia of the affected area. In the negative control group, MI recovery was extremely slow: by day 14, the value reached only 5.4 ± 0.4 perf. units, and by day 28 it was 6.8 ± 0.5 perf. units, remaining statistically significantly lower than the intact level (*p* < 0.05). In the positive control group, the dynamics were slightly better: on the 14th day, MI = 6.3 ± 0.4 perf. Units; on the 28th day, it was 7.8 ± 0.6 perf. units. The most pronounced improvement was recorded in the experimental group (CC-PMMA–pectin-glycerin film): already on the 7th day, MI reached 5.7 ± 0.4 perf. units (versus 5.6 ± 0.4 perf. units in the negative control, *p* < 0.05), and by day 28, it was 9.1 ± 0.9 perf. units, which had no statistically significant differences from intact animals (*p* > 0.05). Thus, the use of the developed film contributed to the almost complete normalization of tissue perfusion by the end of the observation.

Spectral analysis of blood flow fluctuations allowed us to assess the contribution of active and passive regulatory mechanisms.

Endothelial oscillations (E, range 0.01–0.08 Hz, Figure 6b) reflect the local production of nitric oxide and other vasoactive factors by the endothelium. In intact animals, the amplitude of E was 12.8 ± 1.1 conv. units. After the burn, endothelial activity increased in all groups (up to 15.8 ± 1.4 conv. units on day 0). In the negative control, E recovery was slow and did not reach the norm by the 28th day (13.1 ± 1.2 conv. units, *p* < 0.05). In the experimental group, endothelial function recovered faster, and on the 28th day it was 12.9 ± 1.1 conv. units (*p* > 0.05 compared with intact). This indicates early activation of endothelium-dependent vasodilation under the influence of the film.

Neurogenic oscillations (N, 0.08–0.2 Hz, Figure 6c) characterize the sympathetic innervation of blood vessels. In intact animals, the amplitude was N = 9.2 ± 0.8 conv. units. The burn led to a significant increase in neurogenic tone (11.1 ± 1.0 conv. units on day 0), which indicated a pronounced sympathetic spasm. In the negative control, the amplitude of N remained elevated up to day 21 (10.6 ± 0.9 conv. units). In the experimental group, the decrease occurred by the 14th day (10.3 ± 0.9 conv. units), and by the 28th day the indicator decreased to 10.1 ± 0.8 conv. units. This indicates a rapid relief of sympathetically mediated vasoconstriction.

Myogenic oscillations (M, 0.2–0.7 Hz, Figure 6d) reflect the tone of the smooth muscles of the arterioles. In intact animals, the amplitude is M = 8.2 ± 0.8 conv. units. On the first day after the burn, its growth was noted (up to 11.9± 1.1 conv. units) due to myogenic spasm. In the negative control, myogenic activity remained elevated until day 21 (10.7 ± 0.9 conv. units). In the group with film, on the 14th day, the amplitude of M decreased to 10.5 ± 0.9 conv. units, and by the 28th day, it decreased to 8.8 ± 0.7 conv. units. This indicates the restoration of normal contractile activity of the arterioles.

Passive oscillations (respiratory R, 0.7–2 Hz, and cardiac C, 2–5 Hz, Figure 6e,f), reflecting central hemodynamics, normally have a low amplitude. After the burn, their compensatory increase was observed, which was more pronounced in the negative control. In the experimental group, the amplitudes of R and C approached normal values by day 14, while in the control groups they continued to increase at 21–28 days.

It was found that in the experimental group, normalization of the microcirculatory index and a decrease in the amplitudes of active regulatory components (E, N, M) to the level of intact animals occurred synchronously with the maximum rate of reduction in the burn defect area (Table 2) and complete epithelialization by day 28. This confirms the hypothesis that improving tissue trophism as a result of restoring adequate microcirculation is one of the key mechanisms for accelerated healing when using the developed films.

## 4. Conclusions

Thus, the conditions for obtaining a film with the structure of three-dimensional matrices for wound coatings based on a copolymer of cod collagen, pectin and MMA obtained under conditions of photocatalysis with the RbTe_1.5_W_0.5_O_6_ oxide and glycerin plasticizer were selected. It is important that the films are resistant to bacteria, as well as fungi, which is important for the practical use of such wound coatings in non-sterile conditions. In order to assess the stability of the obtained films, their physical and mechanical characteristics were investigated. An increase in the initial polymer content in the solution from 5% to 12% led to a simultaneous increase in tensile strength (from 0.6 MPa to 0.9 MPa) and elongation at break (from 61% to 76%).Tests of wound healing on small animals (rats) using new coatings showed that the use of a wound healing film in burns by day 28 caused normalization of microcirculation and regulatory factors regulating microcirculation according to the results of laser Doppler flowmetry, as well as high-speed reduction in the scalped burn wound area according to planimetry data. This may be due to an improvement in trophic processes in the dermal layers of the skin.

The data of morphological studies indicate complete epithelialization with the formation of scar tissue in all studied groups of animals. The local application of biocidal elastic films based on the cod collagen-pectin-MMA copolymer contributes to a statistically significant restoration of all links of the microcirculatory system (endothelial, neurogenic and myogenic) by the 14th-21st day and complete normalization of perfusion by the 28th day, which is not achieved during the natural course of the wound process and exceeds the effect of commercial collagen coating.

The obtained results of the study of elastic films with biocidal properties are of interest for the treatment of wounds of any etiology, although additional clinical trials are required.

## Figures and Tables

**Table 1:** Biocidal activity of polymer films with the incorporated RbTe_1.5_W_0.5_O_6_ photocatalyst.

Material	Bacterial Growth Inhibition Zone, D (mm)	
	Light	Dark
CC-PMMA-pectin-glycerol	26 (strongly bactericidal)	7 (weakly bactericidal)

**Table 2:** Dynamics of the area (S, cm^2^) of rat skin defects during thermal burn regeneration.

Sample/Time	Day 0	Day 7	Day 14	Day 21	Day 28
CC-PMMA-pectin sponge plate *	21.96 ± 1.02	17.91 ± 0.52	8.21 ± 0.12	2.70 ± 0.04	0 (scar)
CC-PMMA-PEG sponge plate *	22.30 ± 0.72	20.04 ± 0.67	10.43 ± 0.38	3.32 ± 0.05	0.68 ± 0.01
CC-PMMA-pectin-glycerol film	22.50 ± 0.64	16.62 ± 0.23	7.60 ± 0.07	2.11 ± 0.08	0 (scar)

* Data for comparison taken from previous studies [22].

## Data Availability

The original contributions presented in this study are included in the article. Further inquiries can be directed to the corresponding author.
